# Common nutritional/inflammatory indicators are not effective tools in predicting the overall survival of patients with small cell lung cancer undergoing first-line chemotherapy

**DOI:** 10.3389/fonc.2023.1211752

**Published:** 2023-07-27

**Authors:** Huohuan Tian, Guo Li, Wang Hou, Jing Jin, Chengdi Wang, Pengwei Ren, Haoyu Wang, Jie Wang, Weimin Li, Dan Liu

**Affiliations:** ^1^ Department of Respiratory & Critical Care Medicine, West China Hospital, Sichuan University, Chengdu, Sichuan, China; ^2^ Chinese Academy of Medical Sciences (CAMS) Key Laboratory of Translational Research on Lung Cancer, State Key Laboratory of Molecular Oncology, Department of Medical Oncology, National Cancer Center/National Clinical Research Center for Cancer/Cancer Hospital, Chinese Academy of Medical Sciences Peking Union Medical College, Beijing, China

**Keywords:** small cell lung cancer, nutrition, inflammation, biomarkers, overall survival

## Abstract

**Objective:**

Various studies have investigated the predictive significance of numerous peripheral blood biomarkers in patients with small cell lung cancer (SCLC). However, their predictive values have not been validated. This study assessed and evaluated the ability of common nutritional or inflammatory indicators to predict overall survival (OS) in patients with SCLC who received first-line chemotherapy.

**Methods:**

Between January 2008 and July 2019, 560 patients with SCLC were enrolled at the Sichuan University West China Hospital. Eleven nutritional or inflammatory indices obtained before chemotherapy were evaluated. The cutoff values of continuous peripheral blood indices were confirmed through maximally selected rank statistics. The relationship of peripheral blood indices with OS was investigated through univariate and multivariate Cox regression analyses. Harrell’s concordance (C-index) and time-dependent receiver operating characteristic curve were used to evaluate the performance of these indices.

**Results:**

A total of 560 patients with SCLC were enrolled in the study. All the patients received first-line chemotherapy. In the univariate Cox analysis, all indices, except the Naples score, were related to OS. In the multivariate analysis, albumin–globulin ratio was an independent factor linked with prognosis. All indices exhibited poor performance in OS prediction, with the area under the curve ranging from 0.500 to 0.700. The lactic dehydrogenase (LDH) and prognostic nutritional index (PNI) were comparatively superior predictors with C-index of 0.568 and 0.550, respectively. The LDH showed incremental predictive values, whereas the PNI showed diminishing values as survival time prolonged, especially for men or smokers. The LDH with highest sensitivity (0.646) and advanced lung cancer inflammation index (ALI) with highest specificity (0.952) were conducive to identifying death and survival at different time points.

**Conclusion:**

Common inflammatory or nutritional biomarkers are only marginally useful in predicting outcomes in patients with SCLC receiving first-line chemotherapy. Among them, LDH, PNI, and ALI are relatively promising biomarkers for prognosis evaluation.

## Introduction

Small cell lung cancer (SCLC) accounts for approximately 15% of all lung cancer cases, which is characterized by a high growth fraction and widespread metastasis (1). Patients with SCLC have a poor prognosis, with a 5-year survival rate of less than 7% (2).

The Veterans Administration Lung Study Group (VALSG) system and the eighth edition of the American Joint Committee on Cancer TNM classification are widely accepted as the staging system. The National Comprehensive Cancer Network (NCCN) guidelines recommend surgery, followed by adjuvant treatment, for patients with limited-stage cancer at T1-2N0M0 (3). Currently, the standard of care for patients in the extensive stage is chemotherapy or chemotherapy combined with immunotherapy (3). Although cancer stage and treatment strategy are decisive factors for cancer prognosis, weight loss, levels of lactate dehydrogenase, creatinine, and serum sodium were also reported to be associated with the prognosis of chemoradiotherapy-treated locally advanced SCLC (4).

Recent studies have shown that some indices calculated on the basis of peripheral blood cells and biochemical markers can be used to tailor the treatment response or prognosis of with lung cancer (5, 6). These indices are mainly associated with inflammatory response, infection, malnutrition, sarcopenia, or cachexia, which are common complications observed during lung cancer management. Examples of these indices are neutrophil–lymphocyte ratio (NLR), platelet–lymphocyte ratio (PLR), lymphocyte–monocyte ratio (LMR), prognostic nutritional index (PNI), and geriatric nutritional risk index (GNRI) (7, 8). It was reported that NLR and PLR could enhance the prediction accuracy and stability for the prognosis of limited-stage SCLC (9). Furthermore, the high PNI level appears to be an independent beneficial predictor of patients with chemotherapy-treated SCLC (10). According to a prospective analysis, GNRI is linked to treatment response in extensive-stage SCLC (11). Moreover, the controlling nutritional status score (CONUT score) has been indicated as a predictor of recurrence and survival time for patients with SCLC (12). The predictive ability of these biomarkers, however, has not been examined. Furthermore, the optimal index has not been identified. Most importantly, these studies have used a mix of all patients with SCLC who received distinct treatments, which may lead to a considerable bias. Therefore, we conducted this retrospective study to determine and compare the prognostic capability of common inflammatory/nutritional biomarkers in patients with SCLC who received first-line chemotherapy.

## Methods

### Participants

This single-center retrospective study was conducted on patients diagnosed as having SCLC through biopsy at the West China Hospital between January 2008 and July 2019. The study included patients (1) diagnosed as having SCLC through biopsy, (2) whose basic clinical information and data about peripheral blood tests before treatment initiation were available, and (3) who received first-line chemotherapy. We excluded patients (1) whose clinical information and peripheral blood test details were unavailable, (2) peripheral blood tests were after initiation of chemotherapy, and (3) who were lost to follow-up. In total, 560 patients with SCLC who underwent routine in-hospital laboratory tests were included in the study. This study protocol was approved by the West China Hospital Ethics Committee of Sichuan University.

### Clinical information collection and follow-up

To retrieve the fundamental patient data, electronic medical records, including case notes and pathology reports, were analyzed. The retrieved data included age, sex, weight, height, VALSG stage, smoking history, metastasis site, complication, comorbidity, and therapy strategy. The blood biomarkers evaluated at pathological diagnosis or before initiating chemotherapy were neutrophil count (10^9^/L), lymphocyte count (10^9^/L), monocyte count (10^9^/L), platelet count (10^9^/L), albumin (g/L), albumin–globulin ratio (AGR), lactic dehydrogenase (LDH) (U/L), creatinine (μmol/L), cholesterol (mmol/L), neuron-specific enolase (NSE) (U/mL), and carcino embryonic antigen (CEA) (ng/mL). The survival status was determined from the date of the last follow-up in July 2019. The patients were followed up every 3 months by telephone. The outcome was overall survival (OS) time, which was measured from the time that SCLC was diagnosed up until the time of death or the last follow-up.

### Definition and cutoff values of biomarkers

NLR, PLR, and LMR represented the neutrophil–lymphocyte count ratio, the platelet–lymphocyte count ratio, and the lymphocyte–monocyte count ratio in the whole blood, respectively. The PNI was defined as the albumin concentration (g/L) in the whole blood plus five times the total lymphocyte count (10^9^/L). The advanced lung cancer inflammation index (ALI) was defined as body mass index × serum albumin (g/L)/NLR. The GNRI was defined as 1.489 × albumin (g/L) − 41.7 × (actual weight/ideal weight). An ideal weight for men was defined as 0.75 × height (cm) − 62.5, whereas that for women was defined as 0.60 × height (cm) − 40. For patients whose actual weight exceeded the ideal weight, the actual weight/ideal weight was set to 1. The creatinine–cystatin C ratio (ScrCys) was the ratio of serum creatinine and cystatin C. The Naples score was calculated from the NLR, LMR, and albumin and cholesterol levels. The CONUT score was derived on the basis of the serum albumin concentration, total blood cholesterol level, and total peripheral lymphocyte count. The optimal cutoff values of NLR, PLR, LMR, PNI, GNRI, AGR, ScrCys, LDH, NSE, and CEA were determined through the maximally selected rank statistics (13, 14). The cutoff point of Naples and CONUT scores was defined as 2.

### Statistics analysis

The basic clinical characteristics of the included patients were summarized using descriptive statistics. The collinearity among indices was conducted by Pearson correlation analysis. The optimal cutoff values of NLR, PLR, LMR, ALI, PNI, GNRI, AGR, ScrCys, LDH, NSE, and CEA were determined using Jamovi 2.2.5. The prognostic values of all biomarkers and other clinical characteristics were evaluated through the univariate Cox regression analysis. The most important and significant parameters without collinearity in univariate analysis were further submitted for multivariate Cox regression analysis. The capability to predict OS of indices was assessed using Harrell’s concordance index (C-index), time-dependent area under the curve (t-AUC), sensitivity, specificity, positive predictive value (PPV), and negative predictive value (NPV). All statistical analyses were performed using R software version 3.5.1, and all figures were charted using ggplot2 and GraphPad Prism 8.0.

## Results

### Basic clinical characteristics of patients

**Figure 1:**
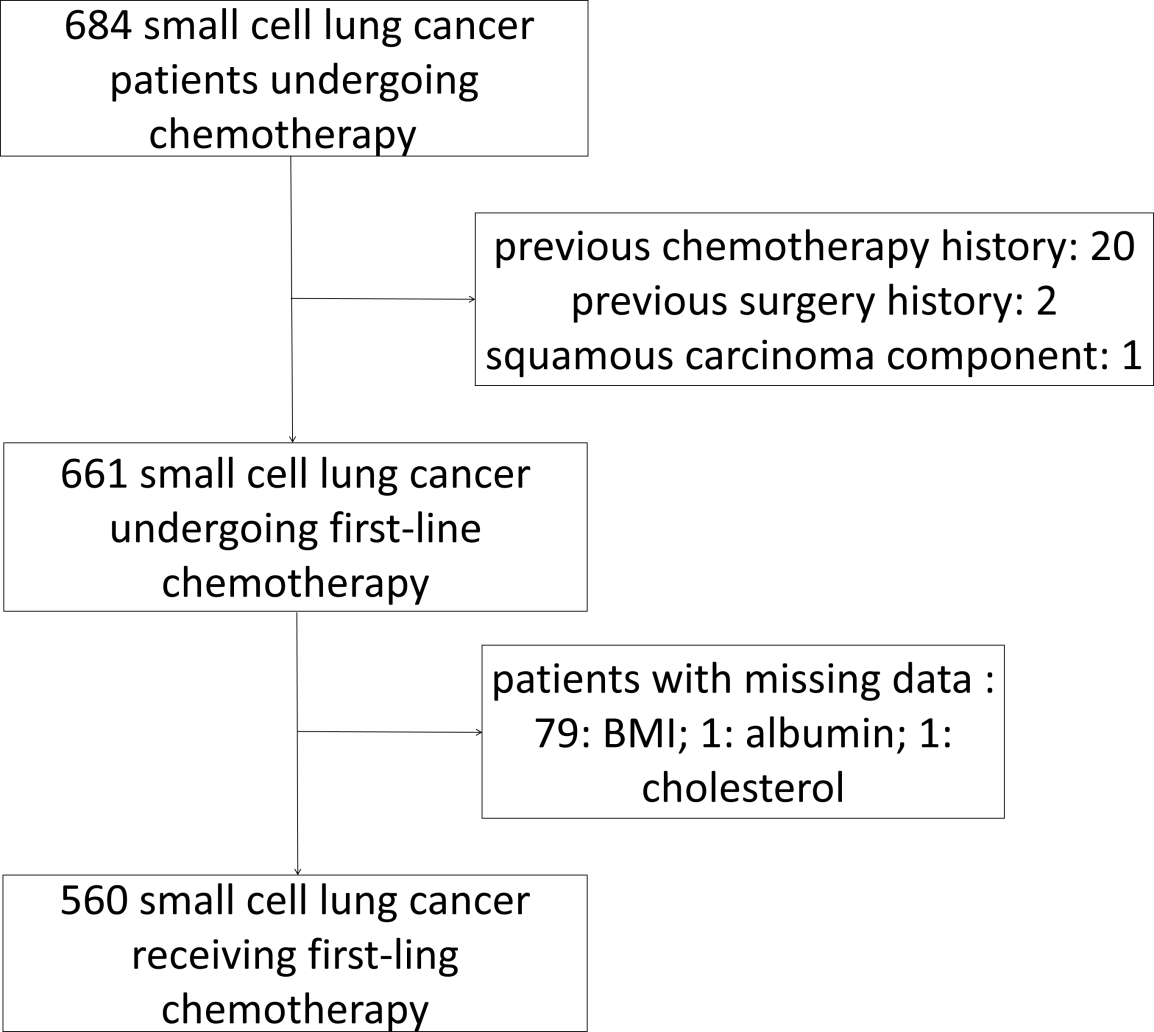
Flowchart of the selection of study population and exclusion criteria.

**Figure 2:**
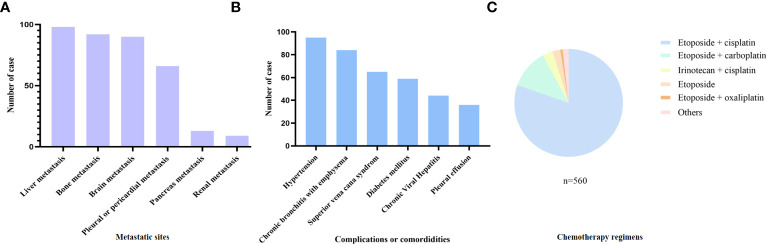
Baseline clinical characteristics of the included patients. **(A)** Metastasis sites, **(B)** complications or comorbidities, and **(C)** chemotherapy regimens of the included patients.

In total, 560 patients with SCLC who met the inclusion and exclusion criteria were included (**Figure 1**). Patients’ demographic characteristics are presented in **Table 1** and **Supplementary Table 1**. The mean age at diagnosis was 57 years (range: 28–79 years), and the majority of the patients were men and smokers, which was consistent with the substantial evidence (15). Approximately 67.7% of the patients had evolved to the extensive stage during enrollment. Pretreatment NLR, PLR, LMR, PNI, GNRI, AGR, ALI, ScrCys, LDH, NSE, and CEA were described as continuous variables, whereas Naples and CONUT scores were described as categorical variables (**Table 1**). Liver, bone, and brain metastases were the most common metastatic sites. A total of 11.6% of patients were complicated with superior vena cava syndrome and 6.4% of patients suffered from pleural or pericardial effusion at diagnosis. Hypertension, diabetes mellitus, and chronic bronchitis with emphysema were the most common comorbidities. Nearly all the patients (97.5%) underwent platinum-based chemotherapy (**Figure 2**, **Supplementary Table S1**). A total of 206 patients received thoracic radiotherapy and 49 patients received prophylactic cranial irradiation combined with first-line chemotherapy (**Supplementary Table S1**). Median OS was 393 days, and median follow-up time was 1941 days.

**Table 1:** Baseline characteristics of all study participants.

Variables	All (n = 560)
**Age (year), Mean (SD)**	57.04 (9.51)
Sex, n (%)	
**Male**	425 (75.9)
**Female**	135 (24.1)
Smoking, n (%)	
**Yes**	395 (70.5)
**No**	165 (29.5)
**BMI(Kg/m2), Mean (SD)**	23.02 (3.18)
Stage, n (%)	
**Limited stage**	181 (32.3)
**Extensive stage**	379 (67.7)
**ALI, Median (IQR)**	307.55 (208.04, 450.10)
**PNI, Mean (SD)**	48.29 (5.91)
**GNRI, Median (IQR)**	100.52 (95.27, 105.58)
**ScrCys, Median (IQR)**	73.66 (65.81, 83.37)
**AGR, Median (IQR)**	1.48 (1.28, 1.67)
**NLR, Median (IQR)**	3.01 (2.17, 4.14)
**PLR, Median (IQR)**	138.93 (98.04, 191.34)
**LMR, Median (IQR)**	3.45 (2.46, 4.61)
Naples score, n (%)	
≤**2**	281 (50.2)
**>2**	279 (49.8)
CONUT score, n (%)	
≤**2**	378 (67.5)
**>2**	183 (32.7)
**LDH (U/L), Median (IQR)**	210 (173,275)
**NSE (U/mL), Median (IQR)**	44.92 (23.25,90.55)
**CEA (ng/mL), Median (IQR)**	3.62 (1.97,8.79)

NLR, neutrophil–lymphocyte ratio; PLR, platelet–lymphocyte ratio; LMR, lymphocyte–monocyte ratio; PNI, prognostic nutritional index; ALI, advanced lung cancer inflammation index; GNRI, geriatric nutritional risk index; ScrCys, creatinine–cystatin C ratio; CONUT score, controlling nutritional status score; AGR, albumin-globulin ratio; LDH, lactic dehydrogenase; NSE, neuron-specific enolase; CEA, carcino embryonic antigen. SD, standard deviation; IQR, interquartile range.

### Determination of the optimal cutoff and prognostic values of indices

**Figure 3:**
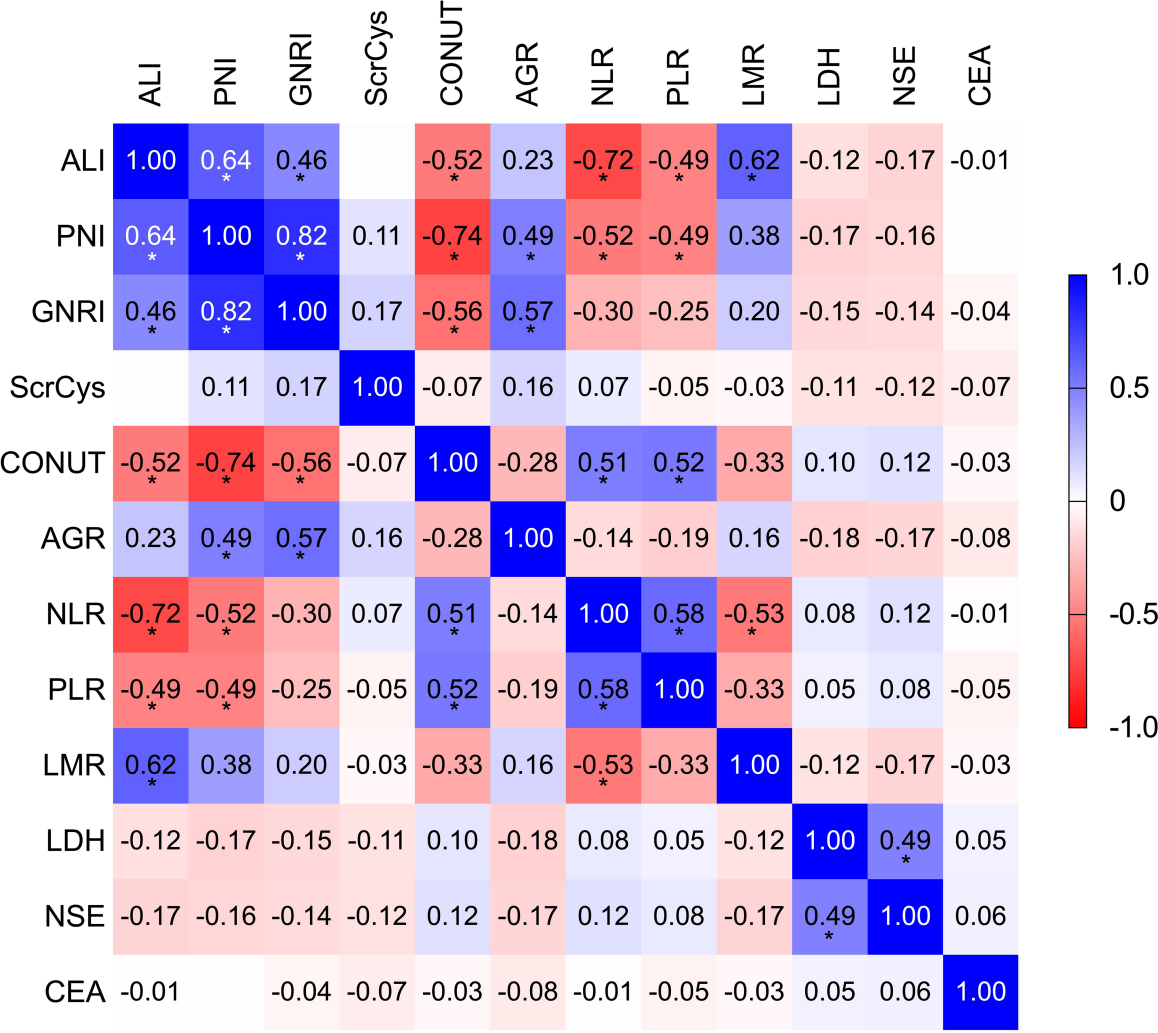
The correlation analysis among all the indices. *P* < 0.05 was labeled as *.

NLR, PLR, LMR, PNI, GNRI, ALI, AGR, ScrCys, LDH, NSE, and CEA had appropriate cutoff values of 3.56, 143.84, 3.50, 45.15, 98.58, 159.04, 1.34, 62.79, 204.00, 22.35, and 5.36, respectively (**Supplementary Figure S1**). These cutoff values were close to those reported in previous investigations. All biomarkers were dichotomized into high or low groups according to the corresponding cutoff values. The univariate analysis was conducted to reveal the unadjusted relationship between biomarkers and OS. Except for the Naples score, all other inflammatory/nutritional biomarkers were associated with OS (**Table 2**). Because candidate indices were derived from common blood parameters, the correlation analysis was performed. ALI, PNI, GNRI, CONUT, NLR, PLR, and LMR showed a significant correlation reciprocally with r coefficient > 0.40 and *P-*value < 0.05 (**Figure 3**; **Supplementary Table S2**). Therefore, ALI, AGR, ScrCys, NSE, and CEA were eventually incorporated into multivariate analysis, which were not correlated with each other. In the multivariate Cox analysis, low AGR that indicated poor physical nutrition or immune state remained as an independent risk factor for survival after adjusted by sex, smoking, stage, metastasis sites, complications, TRT, PCI, and tumor biomarkers (HR, 1.25; 95% CI, 1.02–1.54; *P*-value, 0.033).

**Table 2:** Univariate and multivariate analyses in relation to the patient’s overall survival.

Variables	Univariate analysis			Multivariate analysis		
	HR	95% CI	*P* value	HR	95% CI	*P-*value
Sex (male)	1.44	1.17–1.78	0.001	1.25	0.81–1.92	0.321
Age	1.01	1.00–1.02	0.117			
Smoking (yes)	1.43	1.18–1.74	<0.001	1.22	0.82–1.81	0.329
Stage (LS)	0.56	0.46–0.69	<0.001	0.92	0.71–1.19	0.508
BMI	0.97	0.95–1.00	0.076			
Pleura or pericardium metastasis (yes)	1.29	0.99–1.69	0.064			
Brain metastasis (yes)	1.42	1.13–1.79	0.003	1.04	0.79–1.37	0.762
Bone metastasis (yes)	1.26	1.00–1.60	0.052			
Liver metastasis (yes)	1.68	1.34–2.11	<0.001	1.14	0.88–1.49	0.322
Adrenal gland metastasis (yes)	1.26	0.97–1.64	0.081			
Superior vena cava syndrome (yes)	1.44	1.10–1.87	0.007	1.38	1.02–1.87	0.035
Pleural or pericardial effusion (yes)	1.50	1.07–2.11	0.019	0.97	0.66–1.43	0.878
CB with emphysema (yes)	1.49	1.17–1.90	0.001	1.12	0.85–1.48	0.401
Chronic viral hepatitis (yes)	0.83	0.60–1.16	0.284			
Hypertension (yes)	0.87	0.68–1.10	0.247			
Diabetes mellitus (yes)	1.05	0.79–1.40	0.726			
Thoracic radiotherapy (TRT) (yes)	0.44	0.36–0.54	<0.001	0.58	0.45–0.75	<0.001
PCI (yes)	0.51	0.36–0.73	<0.001	0.64	0.44–0.94	0.021
EP vs. EC	1.07	0.81–1.42	0.617			
LDH (low)	0.63	0.53–0.75	<0.001			
NSE (low)	0.56	0.44–0.70	<0.001	0.75	0.58–0.96	0.022
CEA (low)	0.66	0.54–0.79	<0.001	0.72	0.58–0.88	0.002
NLR (low)	0.74	0.62–0.89	0.001			
PLR (low)	0.76	0.64–0.91	0.003			
LMR (low)	1.35	1.13–1.62	0.001			
Naples score (≤2)	0.92	0.77–1.10	0.345			
ALI (low)	1.71	1.34–2.20	<0.001	1.28	0.96–1.72	0.091
PNI (low)	1.50	1.25–1.82	<0.001			
GNRI (low)	1.41	1.18–1.69	<0.001			
ScrCys (low)	1.32	1.05–1.65	0.018	1.27	0.98-1.65	0.071
CONUT score (≤2)	0.80	0.67–0.97	0.022			
AGR (low)	1.44	1.20–1.72	<0.001	1.25	1.02–1.54	0.033

LS, limited stage; BMI, body mass index; CB, chronic bronchitis; EP, etoposide + cisplatin; EC, etoposide + carboplatin; ScrCys, creatinine–cystatin C ratio; PCI, prophylactic cranial irradiation.

### Prognostic predictive performance of inflammatory and nutritional indicators

**Figure 4:**
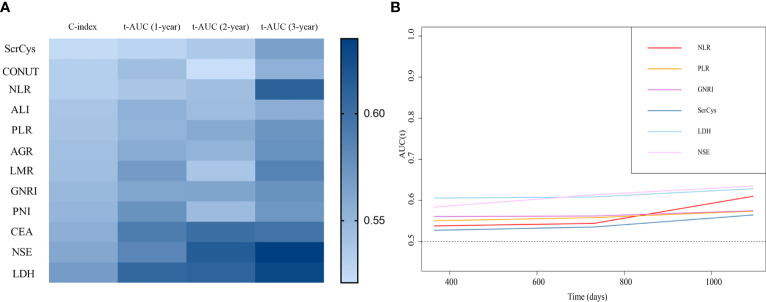
Prognostic predictive performance of the biomarkers. **(A)** C-index and time-dependent AUC at 1-, 2-, and 3-year survival of all the indices. **(B)** Biomarkers showed better prognostic predictive value for long-term survival.

The prognostic predictive performance of indices was assessed using the C-index and t-AUC. As shown in **Table 3** and **Figure 4A**, common inflammation- and nutrition-based indices showed inferior C-index to conventional tumor biomarkers such as NSE and CEA, which reflected their limited values for prognostic prediction (**Table 3**, **Figure 4A**). Among them, LDH and PNI were the relatively desirable indices with the highest C-index of 0.568 and 0.550, respectively. Notably, most of the aforementioned biomarkers exhibited increased predictive capabilities in long-term survival (**Figure 4B**). The LDH was the most valuable indices for 3-year survival with AUC of 0.629. Otherwise, the sensitivity, specificity, PPV, and NPV of all the biomarkers were analyzed. Except for NSE, the LDH showed a highest sensitivity (a high true positive fraction of death) and NPV, whereas ALI showed a highest specificity (a low false positive fraction of death) and PPV at different time points (**Tables 4**, **5**; **Supplementary Tables S3, S4**).

**Table 3:** Prognostic predictive performance of all the biomarkers.

Variables	C-index	1-year AUC	2-year AUC	3-year AUC
NLR	0.532	0.538 (0.498–0.578)	0.544 (0.500–0.589)	0.610 (0.564–0.657)
PLR	0.540	0.551 (0.498–0.578)	0.558 (0.500–0.589)	0.574 (0.564–0.657)
LMR	0.544	0.57 (0.529–0.611)	0.538 (0.490–0.586)	0.586 (0.529–0.642)
ALI	0.539	0.553 (0.524–0.582)	0.545 (0.517–0.573)	0.555 (0.527–0.583)
GNRI	0.548	0.561 (0.520–0.602)	0.562 (0.517–0.608)	0.575 (0.522–0.628)
ScrCys	0.523	0.528 (0.500–0.560)	0.536 (0.501–0.570)	0.565 (0.532–0.598)
CONUT	0.532	0.544 (0.505–0.583)	0.521 (0.476–0.565)	0.553 (0.502–0.603)
AGR	0.543	0.557 (0.517–0.597)	0.551 (0.507–0.595)	0.576 (0.526–0.626)
PNI	0.550	0.575 (0.537–0.613)	0.546 (0.504–0.588)	0.572 (0.525–0.620)
LDH	0.568	0.606 (0.565–0.647)	0.609 (0.562–0.656)	0.629 (0.574–0.684)
NSE	0.560	0.584 (0.548–0.620)	0.614 (0.567–0.661)	0.635 (0.577–0.694)
CEA	0.555	0.591 (0.550–0.631)	0.601 (0.559–0.643)	0.597 (0.549–0.646)

t-AUC, the time-dependent area under ROC; ScrCys, creatinine–cystatin C ratio.

**Table 4:** Sensitivity of all the biomarkers to identify mortality risk at different time points.

Indices	Sensitivity (1 year)	Sensitivity (2 years)	Sensitivity (3 years)
NLR	0.401	0.386	0.4
PLR	0.525	0.501	0.493
LMR	0.584	0.532	0.541
ALI	0.195	0.163	0.157
PNI	0.385	0.331	0.335
GNRI	0.463	0.429	0.424
ScrCys	0.214	0.201	0.201
CONUT	0.374	0.338	0.344
AGR	0.409	0.376	0.379
LDH	0.646	0.587	0.579
NSE	0.850	0.817	0.809
CEA	0.450	0.404	0.390

**Table 5:** Specificity of all the biomarkers at different time points.

Indices	Specificity (1 year)	Specificity (2 years)	Specificity (3 years)
NLR	0.675	0.703	0.824
PLR	0.576	0.616	0.655
LMR	0.556	0.543	0.631
ALI	0.911	0.928	0.952
PNI	0.765	0.761	0.810
GNRI	0.659	0.696	0.726
ScrCys	0.841	0.870	0.930
CONUT	0.715	0.703	0.762
AGR	0.705	0.725	0.774
LDH	0.566	0.630	0.679
NSE	0.318	0.411	0.462
CEA	0.731	0.797	0.805

### Subgroup analysis of the predictive value of inflammation/nutrition-based biomarkers

We further assessed the values of the inflammation/nutrition-based biomarkers for SCLC subgroups. All the biomarkers remained poor predictive performance in all the subgroups with C-index in the 0.500–0.600 range. Compared with other indices, the LDH, PNI, and AGR were the most prominent predictors for men and smokers. These groups of patients accounted for the majority of participants. Similarly, the LDH showed increasing predictive values, whereas the PNI showed diminishing values in this population as the survival time prolonged. The LDH was significant predictor for women or non-smokers (**Supplementary Tables S5–S8**).

## Discussion

Currently, numerous nutrition- and inflammation-based indices have been shown to serve as prognostic markers for SCLC. We verified and contrasted the prognostic predictive efficiency of these indices in a sizable cohort. The AGR was an independent factor for survival after adjusted for clinical information. However, all these prognostic biomarkers or scores had C-index and t-AUC ranging from 0.500 to 0.700, which indicated that these peripheral blood parameters were not effective enough in prognosis prediction. The LDH and PNI were relatively valuable with C-index of 0.568 and 0.550, respectively. The LDH and ALI showed highest sensitivity (negative predictive value) and specificity (positive predictive value), respectively, indicating a promising significance of predicting survival and death at different time points. 

As a canonical tumor and inflammation biomarker, LDH that had collinearity relationship with NSE showed superior performance in prognostic prediction in this study, especially for long-term survival. Previous studies have confirmed that the LDH level was an independent risk for chemotherapy effect and OS of SCLC (16, 17). Cancer cells metabolizes 10-fold glucose through the glycolysis pathway rather than through mitochondrial respiration to produce energy known as the Warburg effect, in which LDH catalyzes the transformation of pyruvate to lactic acid (18). It is also associated with tumor burden, tumor immunogenicity, and activation of oncogenic signaling pathways (19). Although a partial list of innovative prognostic scores were proposed present study demonstrated the relative superiority of LDH and NSE for patients with SCLC undergoing first-line chemotherapy. Meanwhile, the LDH and NSE with highest sensitivity (highest negative predictive value) may be conducive to predicting survival probability at different time points.

Second to LDH and NSE, the PNI was also manifested as a valuable index for prognostic evaluation, especially for short-term survival in smokers or men subgroups. A pooled analysis including 4,164 patients with SCLC suggested that low PNI was correlated with decreased OS in SCLC (20). The study also illustrated that Eastern Cooperative Oncology Group (ECOG) performance status (PS) ≥2, extensive stage, and PCI were influencing factors for PNI. Another study presented the efficacy of the PNI for survival prognostication in patients with SCLC treated with platinum-based chemotherapy with an AUC of 0.564, which resembled our result with a C-index of 0.550 (10). Albumin is involved in the transportation of fatty acids, cholesterol, metal ions, therapeutic agents, and antioxidant effects. Reduced albumin level signifies multiple physiological function impairment, which negatively affects outcomes. Patients with low albumin levels, a sign of poor nutritional status, are prone to experiencing cachexia or infection. Lymphocyte deficiency symbolizes host immunosuppression, which favors tumor development and pathogen aggression. A study suggested that patients with SCLC who received radiotherapy showed aberrant alteration of circulating lymphocyte subsets (21). It was also demonstrated that precursors in peripheral blood could contribute to terminal tumor-infiltrating CD8+ T lymphocytes (22). Because of the correlation between ECOG-PS and PNI, their efficacy for prognosis assessment should have been compared. Similar to PNI, the AGR also reflects physical nutritional and immune status. It could serve as a simple prognostic marker in patients with SCLC (23), which was proved in our study, especially for smokers and men subgroups. Albumin synthesis decreases in response to the production of some inflammatory factors involved in tumor immunity and the acute phase response, such as tumor necrosis factor, interleukin-6, and C-reactive protein (CRP) (24–26). In contrast to the trend in albumin variability, globulin synthesis increases with the accumulation of CRP and other acute-phase reactants (27). Consequently, lower AGR is associated with worse survival in patients with advanced malignancy and a high risk of tumor recurrence after undergoing complete resection (23, 28, 29).

As opposed to LDH, the ALI exhibited outstanding specificity (positive predictive value) at different time points. That is to say, a low level of ALI could be used to predict death probability. Accumulating investigations tend to concentrate on the importance of neutrophil, lymphocyte, and albumin. The ALI is exactly a composite index combining these parameters. Neutrophil infiltration and neutrophils extracellular traps in tumor microenvironment have been demonstrated to facilitate tumor progression and metastasis (30, 31). Tumor cells in return can produce granulocyte colony-stimulating factor, which skews the balance of neutrophil retention and release in bone marrow causing alteration of circulation neutrophils counts (32). A recent research conceded that ALI was the optimal inflammatory biomarkers of overall survival in patients with lung cancer (14). However, they did not report the sensitivity and specificity of ALI. We observed a wide range of cutoff points among various relevant studies, which will have profound impact on sensitivity and specificity of the biomarker (33–35). Thus, the excellent specificity in our study should be validated in an external cohort.

Regardless, because of the critical role of metabolism and inflammation in cancer occurrence and management, nutrition- or inflammation-based biomarkers can be employed as adjuvant measurements in prognostic estimation. However, their performance is poor and inferior to conventional tumor biomarkers. Actually, similar outcomes were also described in other available studies (14, 35). Because the peripheral biomarkers are not entirely identical to that in tumor microenvironment, and patients’ clinical manifestations are volatile, their application in clinical practice should be prudent.

Our study has some limitations. First, data of more completed variables, including serum CRP, and procalcitonin, were not acquired during baseline data collection and processing, leading to the absence of some crucial biomarkers such as CRP/albumin. Moreover, TNM stage, ECOG-PS, response evaluation, and later-line treatment after chemotherapy resistance should have been included as clinical variables. In addition, according to the NCCN guideline, chemoimmunotherapy is preferred as the first-line systemic therapy for patients with extensive-stage SCLC with an ECOG performance score of 0–2. Hence, studies involving a population undergoing novel treatment strategies are warranted. Finally, this was a retrospective study without independent external validation and, thus, inevitably involves considerable bias.

## Conclusion

Common inflammatory or nutritional indices are only marginally useful in predicting outcomes in patients with SCLC receiving first-line chemotherapy. The aforementioned variables should be prudently used as adjuvant measurements in clinical practice. Among them, the LDH and PNI are relatively superior. The LDH and ALI are promising biomarkers to identify death and live patients at different time points.

## Data availability statement

The raw data supporting the conclusions of this article will be made available by the authors, without undue reservation.

## Author contributions

Conception and design: HT, GL, WH, and JJ. Administrative support: DL, WL, and JW. Provision of the study materials orpatients: CW and PR. Collection and assembly of the data: HT, GL, and WH. Data analysis and interpretation: HT and JJ. Review of the manuscript: GL, HW, and JW. Manuscript writing: All authors. Final approval of manuscript: All authors.

## Supplementary material

Supplementary Figure 1 Determination cutoff points of all the biomarkers.

## References

[1] Siegel RL Miller KD Jemal A. Cancer statistics, 2020. CA: Cancer J Clin (2020) 70(1):7–30. doi: 10.3322/caac.21590 31912902

[2] Zhang B Birer SR Dvorkin M Shruti J Byers L. New therapies and biomarkers: are we ready for personalized treatment in small cell lung cancer? Am Soc Clin Oncol Educ book Am Soc Clin Oncol Annu Meeting (2021) 41:1–10. doi: 10.1200/EDBK_320673 33979194

[3] Ganti AKP Loo BW Bassetti M Blakely C Chiang A D'Amico TA. Small cell lung cancer, version 2.2022, NCCN clinical practice guidelines in oncology. J Natl Compr Cancer Network JNCCN (2021) 19(12):1441–64. doi: 10.6004/jnccn.2021.0058 PMC10203822 34902832

[4] Dingemans AC Früh M Ardizzoni A Besse B Faivre-Finn C Hendriks LE. Small-cell lung cancer: ESMO clinical practice guidelines for diagnosis, treatment and follow-up(☆). Ann Oncol Off J Eur Soc Med Oncol (2021) 32(7):839–53. doi: 10.1016/j.annonc.2021.03.207 PMC9464246 33864941

[5] Cortellini A Ricciuti B Borghaei H Naqash AR D'Alessio A Fulgenzi CAM. Differential prognostic effect of systemic inflammation in patients with non-small cell lung cancer treated with immunotherapy or chemotherapy: a *post hoc* analysis of the phase 3 OAK trial. Cancer (2022) 128(16):3067–79. doi: 10.1002/cncr.34348 PMC11497250 35727053

[6] Wang J Li H Xu R Lu T Zhao J Zhang P. NLR, PLR and d-dimer are associated with clinical outcome in lung cancer patients treated with surgery. BMC Pulmonary Med (2022) 22(1):104. doi: 10.1186/s12890-022-01901-7 35337299 PMC8957174

[7] Ruan GT Yang M Zhang XW Song MM Hu CL Ge YZ. Association of systemic inflammation and overall survival in elderly patients with cancer cachexia - results from a multicenter study. J Inflamm Res (2021) 14:5527–40. doi: 10.2147/JIR.S332408 PMC8558830 34737602

[8] Karayama M Inoue Y Yasui H Hozumi H Suzuki Y Furuhashi K. Association of the geriatric nutritional risk index with the survival of patients with non-small-cell lung cancer after platinum-based chemotherapy. BMC Pulmonary Med (2021) 21(1):409. doi: 10.1186/s12890-021-01782-2 34895201 PMC8665565

[9] Chen C Yang H Cai D Xiang L Fang W Wang R. Preoperative peripheral blood neutrophil-to-lymphocyte ratios (NLR) and platelet-to-lymphocyte ratio (PLR) related nomograms predict the survival of patients with limited-stage small-cell lung cancer. Trans Lung Cancer Res (2021) 10(2):866–77. doi: 10.21037/tlcr-20-997 PMC7947425 33718028

[10] Jin S Cao S Xu S Wang C Meng Q Yu Y. Clinical impact of pretreatment prognostic nutritional index (PNI) in small cell lung cancer patients treated with platinum-based chemotherapy. Clin Respir J (2018) 12(9):2433–40. doi: 10.1111/crj.12925 30074685

[11] Lee GW Go SI Kim DW Kim HG Kim JH An HJ. Geriatric nutritional risk index as a prognostic marker in patients with extensive-stage disease small cell lung cancer: results from a randomized controlled trial. Thorac Cancer (2020) 11(1):62–71. doi: 10.1111/1759-7714.13229 31707767 PMC6938749

[12] Yılmaz A Tekin SB Bilici M Yılmaz H. The significance of controlling nutritional status (CONUT) score as a novel prognostic parameter in small cell lung cancer. Lung (2020) 198(4):695–704. doi: 10.1007/s00408-020-00361-2 32424800

[13] Liu T Liu C Deng L Song M Lin S Shi H. The prognostic effect of sixteen malnutrition/inflammation-based indicators on the overall survival of chemotherapy patients. Front Immunol (2023) 14:1117232. doi: 10.3389/fimmu.2023.1117232 36875131 PMC9978470

[14] Song M Zhang Q Song C Liu T Zhang X Ruan G. The advanced lung cancer inflammation index is the optimal inflammatory biomarker of overall survival in patients with lung cancer. J Cachexia Sarcopenia Muscle (2022) 13(5):2504–14. doi: 10.1002/jcsm.13032 PMC9530543 35833264

[15] Wang Q Gümüş ZH Colarossi C Memeo L Wang X Kong CY. SCLC: epidemiology, risk factors, genetic susceptibility, molecular pathology, screening, and early detection. J Thorac Oncol Off Publ Int Assoc Study Lung Cancer (2023) 18(1):31–46. doi: 10.1016/j.jtho.2022.10.002 PMC10797993 36243387

[16] He M Chi X Shi X Sun Y Yang X Wang L. Value of pretreatment serum lactate dehydrogenase as a prognostic and predictive factor for small-cell lung cancer patients treated with first-line platinum-containing chemotherapy. Thorac Cancer (2021) 12(23):3101–9. doi: 10.1111/1759-7714.13581 PMC8636211 34725930

[17] Zhang X Guo M Fan J Lv Z Huang Q Han J. Prognostic significance of serum LDH in small cell lung cancer: a systematic review with meta-analysis. Cancer Biomarkers section A Dis Markers (2016) 16(3):415–23. doi: 10.3233/CBM-160580 PMC13016485 27062698

[18] Koppenol WH Bounds PL Dang CV. Otto Warburg's contributions to current concepts of cancer metabolism. Nat Rev Cancer (2011) 11(5):325–37. doi: 10.1038/nrc3038 21508971

[19] Claps G Faouzi S Quidville V Chehade F Shen S Vagner S. The multiple roles of LDH in cancer. Nat Rev Clin Oncol (2022) 19(12):749–62. doi: 10.1038/s41571-022-00686-2 36207413

[20] Jiang AM Zhao R Liu N Ma YY Ren MD Tian T. The prognostic value of pretreatment prognostic nutritional index in patients with small cell lung cancer and it's influencing factors: a meta-analysis of observational studies. J Thorac Disease (2020) 12(10):5718–28. doi: 10.21037/jtd-20-1739 PMC7656400 33209404

[21] Li H Yu H Lan S Zhao D Liu Y Cheng Y. Aberrant alteration of circulating lymphocyte subsets in small cell lung cancer patients treated with radiotherapy. Technol Cancer Res Treat (2021) 20:15330338211039948. doi: 10.1177/15330338211039948 34851203 PMC8649432

[22] Gueguen P Metoikidou C Dupic T Lawand M Goudot C Baulande S. Contribution of resident and circulating precursors to tumor-infiltrating CD8(+) T cell populations in lung cancer. Sci Immunol (2021) 6(55):eabd5778. doi: 10.1126/sciimmunol.abd5778 33514641

[23] Zhou T He X Fang W Zhan J Hong S Qin T. Pretreatment Albumin/Globulin ratio predicts the prognosis for small-cell lung cancer. Medicine (2016) 95(12):e3097. doi: 10.1097/MD.0000000000003097 27015181 PMC4998376

[24] Bankey PE Mazuski JE Ortiz M Fulco JM Cerra FB. Hepatic acute phase protein synthesis is indirectly regulated by tumor necrosis factor. J Trauma (1990) 30(10):1181–7. doi: 10.1097/00005373-199010000-00001 1698990

[25] Pfensig C Dominik A Borufka L Hinz M Stange J Eggert M. A new application for albumin dialysis in extracorporeal organ support: characterization of a putative interaction between human albumin and proinflammatory cytokines IL-6 and TNFα. Artif Organs (2016) 40(4):397–402. doi: 10.1111/aor.12557 26365493

[26] Kim S McClave SA Martindale RG Miller KR Hurt RT. Hypoalbuminemia and clinical outcomes: what is the mechanism behind the relationship? Am Surgeon (2017) 83(11):1220–7. doi: 10.1177/000313481708301123 29183523

[27] Zhang H Zhang B Zhu K Wu C Gao L Sun X. Preoperative albumin-to-globulin ratio predicts survival in patients with non-small-cell lung cancer after surgery. J Cell Physiol (2019) 234(3):2471–9. doi: 10.1002/jcp.26766 30317549

[28] Yao Y Zhao M Yuan D Gu X Liu H Song Y. Elevated pretreatment serum globulin albumin ratio predicts poor prognosis for advanced non-small cell lung cancer patients. J Thorac Disease (2014) 6(9):1261–70. doi: 10.3978/j.issn.2072-1439.2014.07.13DATAHERE PMC4178101 25276368

[29] Jin Y Zhao L Peng F. Prognostic impact of serum albumin levels on the recurrence of stage I non-small cell lung cancer. Clinics (Sao Paulo Brazil) (2013) 68(5):686–93. doi: 10.6061/clinics/2013(05)17 PMC3654299 23778417

[30] Faget J Groeneveld S Boivin G Sankar M Zangger N Garcia M. Neutrophils and snail orchestrate the establishment of a pro-tumor microenvironment in lung cancer. Cell Rep (2017) 21(11):3190–204. doi: 10.1016/j.celrep.2017.11.052 29241546

[31] Mousset A Lecorgne E Bourget I Lopez P Jenovai K Cherfils-Vicini J. Neutrophil extracellular traps formed during chemotherapy confer treatment resistance via TGF-β activation. Cancer Cell (2023) 41(4):757–75.e10. doi: 10.1016/j.ccell.2023.03.008 37037615 PMC10228050

[32] Jablonska J Lang S Sionov RV Granot Z. The regulation of pre-metastatic niche formation by neutrophils. Oncotarget (2017) 8(67):112132–44. doi: 10.18632/oncotarget.22792 PMC5762385 29340117

[33] He X Zhou T Yang Y Hong S Zhan J Hu Z. Advanced lung cancer inflammation index, a new prognostic score, predicts outcome in patients with small-cell lung cancer. Clin Lung Cancer (2015) 16(6):e165–71. doi: 10.1016/j.cllc.2015.03.005 25922292

[34] Hu Z Wu W Zhang X Li P Zhang H Wang H. Advanced lung cancer inflammation index is a prognostic factor of patients with small-cell lung cancer following surgical resection. Cancer Manage Res (2021) 13:2047–55. doi: 10.2147/CMAR.S295952 PMC7924125 33664592

[35] Zhou T Zhao Y Zhao S Yang Y Huang Y Hou X. Comparison of the prognostic value of systemic inflammation response markers in small cell lung cancer patients. J Cancer (2019) 10(7):1685–92. doi: 10.7150/jca.29319 PMC6547997 31205524

