# Supplementary figures and images for: Common nutritional/inflammatory indicators are not effective tools in predicting the overall survival of patients with small cell lung cancer undergoing first-line chemotherapy
Source: Front Oncol. 2023 Jul 27;13:1211752. doi: 10.3389/fonc.2023.1211752 (PMC10421701; doi:10.3389/fonc.2023.1211752)

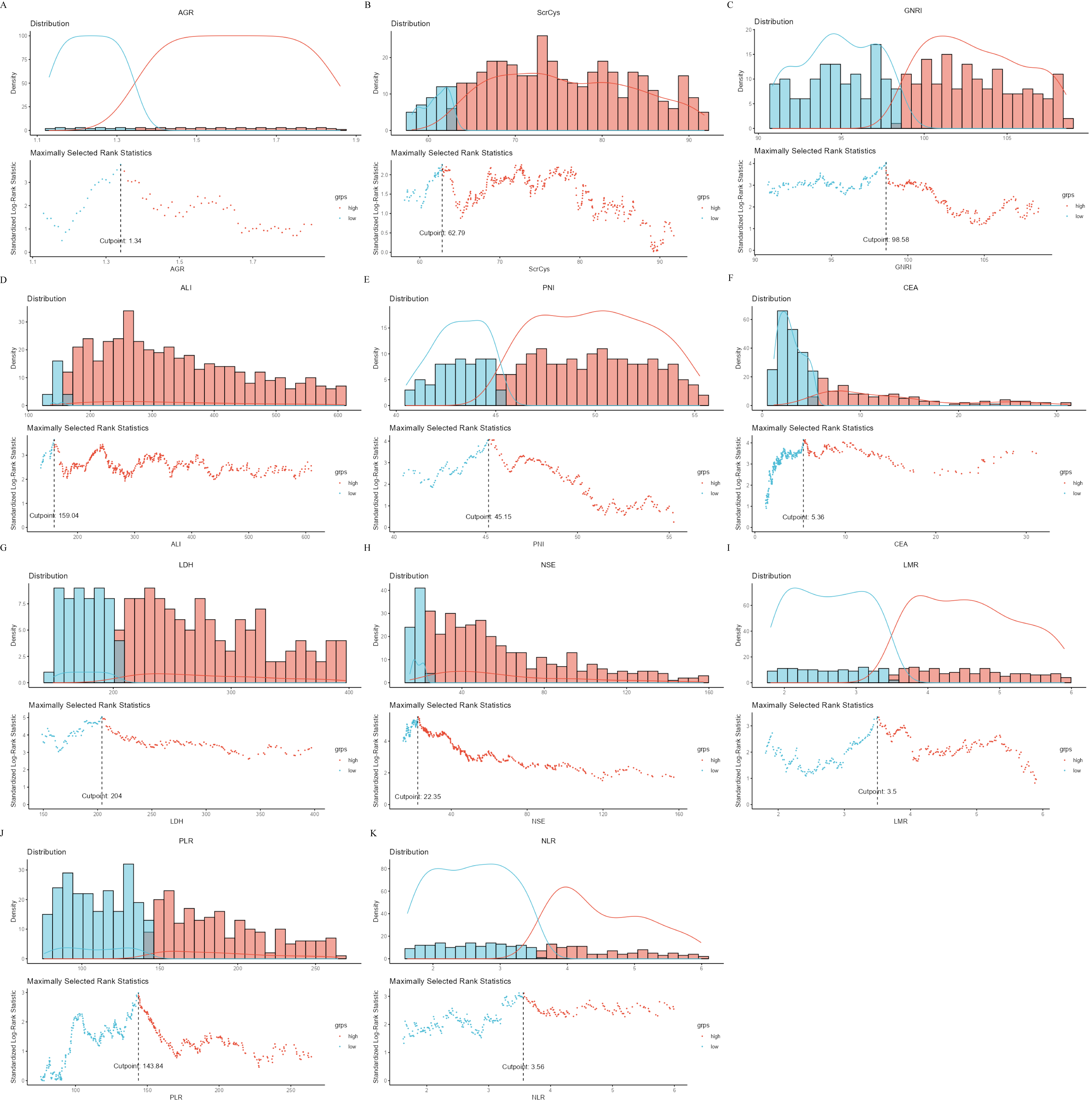

Supplement: Supplementary Figure 1 — Determination cutoff points of all the biomarkers. [file Image_1.tif]
