# Supplementary material for: Common nutritional/inflammatory indicators are not effective tools in predicting the overall survival of patients with small cell lung cancer undergoing first-line chemotherapy
Source: Front Oncol. 2023 Jul 27;13:1211752. doi: 10.3389/fonc.2023.1211752 (PMC10421701; doi:10.3389/fonc.2023.1211752)
Supplement: Supplementary file 2 [file Table_1.docx]

Supplementary Table 1 Baseline characteristics of all study participants.

| Variables | **n (%)** |
| --- | --- |
| Metastasis sites |  |
| Brain metastasis | 90 (16.1) |
| Liver metastasis | 98 (17.5) |
| Bone metastasis | 92 (16.4) |
| Adrenal gland metastasis | 72 (12.9) |
| Pleura or pericardium metastasis | 66 (11.8) |
| Pancreas metastasis | 13 (2.3) |
| Renal metastasis | 9 (1.6) |
| Comorbidities or complications |  |
| Superior vena cava syndrome | 65 (11.6) |
| Pleural or pericardial effusion | 36 (6.4) |
| COPD or Chronic bronchitis with emphysema | 84 (15.0) |
| Chronic viral hepatitis | 44 (7.9) |
| Hypertension | 95 (17.0) |
| Diabetes mellitus | 59 (10.5) |
| chemotherapy regimens |  |
| Etoposide + cisplatin | 450 (80.4) |
| Etoposide + carboplatin | 66 (11.8) |
| Irinotecan + cisplatin | 17 (3.0) |
| Etoposide | 13 (2.3) |
| Etoposide + oxaliplatin | 4 (0.7) |
| others | 10 (1.8) |
| Thoracic radiotherapy (TRT) |  |
| Yes | 206 (36.8) |
| No | 217 (38.8) |
| Unclear | 137 (24.5) |
| Prophylactic cranial irradiation (PCI) |  |
| Yes | 49 (8.8) |
| No | 391 (69.8) |
| Unclear | 120 (21.4) |
| Radiotherapy combined with chemotherapy |  |
| Concurrent radiotherapy | 153 (27.0) |
| Sequential radiotherapy | 108 (19.3) |

Abbreviation: COPD, chronic obstructive pulmonary disease.

Supplementary Table 2 correlation analysis among all the indices.

|  | ALI | PNI | GNRI | ScrCys | CONUT | AGR | NLR | PLR | LMR | LDH | NSE | CEA |
| --- | --- | --- | --- | --- | --- | --- | --- | --- | --- | --- | --- | --- |
| ALI | 1.00 | 0.64 | 0.46 | 0.00 | -0.52 | 0.23 | -0.72 | -0.49 | 0.62 | -0.12 | -0.17 | -0.01 |
| PNI | 0.64 | 1.00 | 0.82 | 0.11 | -0.74 | 0.49 | -0.52 | -0.49 | 0.38 | -0.17 | -0.16 | 0.00 |
| GNRI | 0.46 | 0.82 | 1.00 | 0.17 | -0.56 | 0.57 | -0.30 | -0.25 | 0.20 | -0.15 | -0.14 | -0.04 |
| ScrCys | 0.00 | 0.11 | 0.17 | 1.00 | -0.07 | 0.16 | 0.07 | -0.05 | -0.03 | -0.11 | -0.12 | -0.07 |
| CONUT | -0.52 | -0.74 | -0.56 | -0.07 | 1.00 | -0.28 | 0.51 | 0.52 | -0.33 | 0.10 | 0.12 | -0.03 |
| AGR | 0.23 | 0.49 | 0.57 | 0.16 | -0.28 | 1.00 | -0.14 | -0.19 | 0.16 | -0.18 | -0.17 | -0.08 |
| NLR | -0.72 | -0.52 | -0.30 | 0.07 | 0.51 | -0.14 | 1.00 | 0.58 | -0.53 | 0.08 | 0.12 | -0.01 |
| PLR | -0.49 | -0.49 | -0.25 | -0.05 | 0.52 | -0.19 | 0.58 | 1.00 | -0.33 | 0.05 | 0.08 | -0.05 |
| LMR | 0.62 | 0.38 | 0.20 | -0.03 | -0.33 | 0.16 | -0.53 | -0.33 | 1.00 | -0.12 | -0.17 | -0.03 |
| LDH | -0.12 | -0.17 | -0.15 | -0.11 | 0.10 | -0.18 | 0.08 | 0.05 | -0.12 | 1.00 | 0.49 | 0.05 |
| NSE | -0.17 | -0.16 | -0.14 | -0.12 | 0.12 | -0.17 | 0.12 | 0.08 | -0.17 | 0.49 | 1.00 | 0.06 |
| CEA | -0.01 | 0.00 | -0.04 | -0.07 | -0.03 | -0.08 | -0.01 | -0.05 | -0.03 | 0.05 | 0.06 | 1.00 |

Supplementary Table 3 positive predictive value of all the biomarkers at different time-points.

| Variables | 1-year PPV | 2-year PPV | 3-year PPV |
| --- | --- | --- | --- |
| NLR | 0.512 | 0.794 | 0.915 |
| PLR | 0.513 | 0.795 | 0.87 |
| LMR | 0.530 | 0.777 | 0.871 |
| ALI | 0.649 | 0.872 | 0.935 |
| PNI | 0.582 | 0.808 | 0.89 |
| GNRI | 0.536 | 0.807 | 0.878 |
| ScrCys | 0.534 | 0.824 | 0.93 |
| CONUT | 0.627 | 0.772 | 0.869 |
| AGR | 0.544 | 0.806 | 0.888 |
| LDH | 0.560 | 0.826 | 0.893 |
| CEA | 0.589 | 0.859 | 0.904 |
| NSE | 0.515 | 0.977 | 0.876 |

Supplementary Table 4 negative predictive value of all the biomarkers at different time-points.

| Variables | 1-year NPV | 2-year NPV | 3-year NPV |
| --- | --- | --- | --- |
| NLR | 0.568 | 0.275 | 0.265 |
| PLR | 0.586 | 0.291 | 0.213 |
| LMR | 0.611 | 0.278 | 0.226 |
| ALI | 0.569 | 0.270 | 0.195 |
| PNI | 0.592 | 0.275 | 0.207 |
| GNRI | 0.589 | 0.288 | 0.213 |
| ScrCys | 0.556 | 0.266 | 0.200 |
| CONUT | 0.571 | 0.261 | 0.199 |
| AGR | 0.584 | 0.280 | 0.212 |
| LDH | 0.653 | 0.337 | 0.257 |
| CEA | 0.609 | 0.309 | 0.222 |
| NSE | 0.711 | 0.430 | 0.343 |

Supplementary Table 5 Prognostic predictive performance of all the biomarkers in men subgroup.

| Variables | C-index | 1-year AUC | 2-year AUC | 3-year AUC |
| --- | --- | --- | --- | --- |
| NLR | 0.546 | 0.589 (0.533-0.624) | 0.569 (0.516-0.621) | 0.625 (0.571-0.678) |
| PLR | 0.551 | 0.572 (0.525-0.618) | 0.571 (0.516-0.627) | 0.579 (0.513-0.645) |
| LMR | 0.537 | 0.570 (0.523-0.617) | 0.528 (0.470-0.586) | 0.57 (0.499-0.640) |
| ALI | 0.545 | 0.566 (0.533-0.600) | 0.547 (0.513-0.582) | 0.556 (0.520-0.592) |
| PNI | 0.555 | 0.595 (0.552-0.638) | 0.544 (0.495-0.600) | 0.549 (0.489-0.610) |
| GNRI | 0.556 | 0.581 (0.535-0.627) | 0.583 (0.530-0.638) | 0.584 (0.519-0.649) |
| ScrCys | 0.527 | 0.533 (0.500-0.567) | 0.533 (0.500-0.570) | 0.565 (0.533-0.597) |
| CONUT | 0.542 | 0.562 (0.518-0.606) | 0.538 (0.487-0.590) | 0.575 (0.518-0.632) |
| AGR | 0.555 | 0.574 (0.529-0.618) | 0.579 (0.529-0.628) | 0.600 (0.544-0.655) |
| LDH | 0.566 | 0.605 (0.558-0.651) | 0.612 (0.556-0.668) | 0.638 (0.571-0.704) |
| CEA | 0.555 | 0.593 (0.546-0.639) | 0.600 (0.547-0.653) | 0.592 (0.528-0.655) |
| NSE | 0.551 | 0.565 (0.523-0.607) | 0.607 (0.550-0.665) | 0.647 (0.573-0.720) |

Supplementary Table 6 Prognostic predictive performance of all the biomarkers in smoker subgroup.

| Variables | C-index | 1-year AUC | 2-year AUC | 3-year AUC |
| --- | --- | --- | --- | --- |
| NLR | 0.538 | 0.57 (0.523-0.617） | 0.562 (0.507-0.616) | 0.607 (0.548-0.665) |
| PLR | 0.546 | 0.565 （0.516-0.613） | 0.559 (0.501-0.617) | 0.554 (0.484-0.624) |
| LMR | 0.533 | 0.562 (0.513-0.611) | 0.524 (0.463-0.584) | 0.565 (0.491-0.638) |
| ALI | 0.544 | 0.566 (0.532-0.599) | 0.545 (0.510-0.579） | 0.548 (0.510-0.586) |
| PNI | 0.557 | 0.603 (0.559-0.647) | 0.547 (0.500-0.600) | 0.541 (0.477-0.604) |
| GNRI | 0.556 | 0.583 (0.534-0.631) | 0.581 (0.524-0.637) | 0.579 (0.510-0.647) |
| ScrCys | 0.528 | 0.533 (0.497-0.569) | 0.533 (0.494-0.573) | 0.569 (0.535-0.602) |
| CONUT | 0.547 | 0.570 (0.525-0.615) | 0.541 (0.488-0.594) | 0.564 (0.504-0.624) |
| AGR | 0.561 | 0.583 (0.537-0.629) | 0.585 (0.534-0.636) | 0.607 (0.551-0.664) |
| LDH | 0.564 | 0.605 (0.557-0.653) | 0.604 (0.545-0.663) | 0.62 (0.550-0.690) |
| CEA | 0.560 | 0.599 (0.550-0.647) | 0.611 (0.556-0.666) | 0.601 (0.535-0.667) |
| NSE | 0.548 | 0.565 (0.520-0.609) | 0.602 (0.543-0.662) | 0.625 (0.550-0.701) |

Supplementary Table 7 Prognostic predictive performance of all the biomarkers in women subgroup.

| Variables | C-index | 1-year AUC | 2-year AUC | 3-year AUC |
| --- | --- | --- | --- | --- |
| NLR | 0.493 | 0.398 (0.318-0.478) | 0.493 (0.407-0.578) | 0.586 (0.496-0.676) |
| PLR | 0.541 | 0.525 (0.437-0.612) | 0.570 (0.482-0.657) | 0.601 (0.500-0.702) |
| LMR | 0.549 | 0.542 (0.453-0.631) | 0.533 (0.447-0.620) | 0.594 (0.500-0.687) |
| ALI | 0.515 | 0.494 (0.442-0.546) | 0.533 (0.483-0.582) | 0.544 (0.498-0.590) |
| PNI | 0.548 | 0.515 (0.431-0.600） | 0.555 (0.475-0.635) | 0.626 (0.548-0.703) |
| GNRI | 0.504 | 0.470 (0.387-0.553) | 0.496 (0.413-0.580) | 0.539 (0.446-0.631) |
| ScrCys | 0.541 | 0.5449 (0.465-0.632) | 0.575 (0.500-0.650) | 0.595 (0.518-0.671) |
| CONUT | 0.512 | 0.498 (0.412-0.584) | 0.493 (0.407-0.578) | 0.520 (0.423-0.618) |
| AGR | 0.518 | 0.520 (0.432-0.609) | 0.506 (0.419-0.592) | 0.546 (0.448-0.643) |
| LDH | 0.579 | 0.62 (0.534-0.705) | 0.61 (0.524-0.697) | 0.62 (0.520-0.718) |
| CEA | 0.526 | 0.545 (0.466-0.625) | 0.567 (0.499-0.636) | 0.574 (0.504-0.645) |
| NSE | 0.591 | 0.654 (0.592-0.716) | 0.633 (0.550-0.716) | 0.622 (0.523-0.720) |

Supplementary Table 8 Prognostic predictive performance of all the biomarkers in non-smoker subgroup.

| Variables | C-index | 1-year AUC | 2-year AUC | 3-year AUC |
| --- | --- | --- | --- | --- |
| NLR | 0.52 | 0.457 (0.382-0.533) | 0.517 (0.439-0.595) | 0.622 (0.543-0.701) |
| PLR | 0.555 | 0.547 (0.469-0.625) | 0.587 (0.507-0.668) | 0.635 (0.542-0.728) |
| LMR | 0.56 | 0.572 (0.493-0.651) | 0.545 (0.465-0.625) | 0.607 (0.519-0.700) |
| ALI | 0.528 | 0.519 (0.464-0.574) | 0.547 (0.498-0.596) | 0.566 (0.522-0.610) |
| PNI | 0.543 | 0.509 (0.434-0.583) | 0.549 (0.475-0.622) | 0.632 (0.561-0.702) |
| GNRI | 0.512 | 0.486 (0.412-0.560) | 0.512 (0.436-0.588) | 0.554 (0.470-0.639) |
| ScrCys | 0.527 | 0.535 (0.464-0.605) | 0.557 (0.492-0.622) | 0.573 (0.505-0.642) |
| CONUT | 0.509 | 0.496 (0.419-0.573) | 0.499 (0.420-0.578) | 0.548 (0.458-0.638) |
| AGR | 0.507 | 0.501 (0.423-0.578) | 0.499 (0.420-0.578) | 0.533 (0.442-0.625) |
| CEA | 0.511 | 0.532 (0.464-0.600) | 0.546 (0.483-0.609) | 0.558 (0.490-0.626) |
| NSE | 0.594 | 0.64 (0.584-0.696) | 0.641 (0.565-0.716) | 0.658 (0.565-0.751) |
| LDH | 0.579 | 0.612 (0.534-0.689) | 0.619 (0.541-0.698) | 0.645 (0.555-0.735) |
